# RDEmbed: A Sentence-Embedding Framework for Rare Disease Phenotype Representation and Phenotypic Similarity Analysis

**DOI:** 10.64898/2026.10.16.759399

**Published:** 2026-10-19

**Authors:** Xinghua Wang, Jinlian Wang, Zijian Zhang, Hongfang Liu, Arif Harmanci

## Abstract

Accurate phenotypic characterization of patients with rare diseases is essential for improving diagnosis, patient matchmaking, and gene discovery. Conventional ontology-driven approaches based on the Human Phenotype Ontology (HPO) enable structured phenotype comparison but often limited by the incomplete annotations and reduced ability to capture the contextual information contained in free-text clinical narratives.

We present RDEmbed, a sentence embedding framework that learns patient phenotype representations directly from rare disease case reports for similarity analysis. RDEmbed encodes narrative case reports into high-dimensional vectors to quantify phenotypic similarity. Unlike ontology-based approaches that rely on manually curated HPO annotations, RDEmbed encodes narrative case reports into dense semantic embeddings that capture complex phenotypic relationships from unstructured text. We further introduced retrieval-augmented embedding refinement (RAER), which enhances embedding representations by incorporating external domain knowledge at inference time without task-specific fine-tuning.

RDEmbed was evaluated against HPO-based similarity methods (OWLSim, simGIC) using simulated datasets, published rare disease case reports and a curated case study dataset comprising 156 patient abstracts presenting six rare disease groups. Across the real world datasets and curated cohort. RDEmbed consistently outperformed ontology-based approaches, improving AUROC and AUPRC by more than 5%. Embedding-based representations also produced more distinct disease-specific clustering in t-SNE visualizations and identified twice as many true positive rare disease pairs as ontology-based methods (8 versus 4). Incorporating RAER further improved precision while maintaining high sensitivity compared with zero-shot embeddings.

These results demonstrate that sentence embeddings provide an effective alternative to ontology-based phenotype representations by learning directly from free-text clinical narratives. RDEmbed enables more accurate phenotypic similarity analysis while reducing dependence on manually curated HPO annotations and offers a scalable framework for rare disease diagnosis, patient matchmaking, and gene discovery. RDEmbed is publicly available at https://github.com/xhwang2017/RDEmbed .

